# Genetically-biased fertilization in APOBEC1 complementation factor (*A1cf*) mutant mice

**DOI:** 10.1038/s41598-022-17948-9

**Published:** 2022-08-10

**Authors:** Naoki Hirose, Genevieve Blanchet, Yasuhiro Yamauchi, Abigail C. Snow, Robin Friedman, Carmen Y. Khoo, Christine W. Lary, Monika A. Ward, Joseph H. Nadeau

**Affiliations:** 1 Institute for Biogenesis Research, John A. Burns School of Medicine, University of Hawaii at Manoa, Honolulu, HI USA; 2 Center for Molecular Medicine, Maine Medical Center Research Institute, Portland, ME USA; 3 Ohana Biosciences, Cambridge, MA USA; 4 Center for Outcomes Research, Maine Medical Center Research Institute, Portland, ME USA; 5 Present Address: Dragonfly Therapeutics, Waltham, MA USA

**Keywords:** Genetics, Development

## Abstract

Meiosis, recombination, and gametogenesis normally ensure that gametes combine randomly. But in exceptional cases, fertilization depends on the genetics of gametes from both females and males. A key question is whether their non-random union results from factors intrinsic to oocytes and sperm, or from their interactions with conditions in the reproductive tracts. To address this question, we used in vitro fertilization (IVF) with a mutant and wild-type allele of the *A1cf* (APOBEC1 complementation factor) gene in mice that are otherwise genetically identical. We observed strong distortion in favor of mutant heterozygotes showing that bias depends on the genetics of oocyte and sperm, and that any environmental input is modest. To search for the potential mechanism of the ‘biased fertilization’, we analyzed the existing transcriptome data and demonstrated that localization of *A1cf* transcripts and its candidate mRNA targets is restricted to the spermatids in which they originate, and that these transcripts are enriched for functions related to meiosis, fertilization, RNA stability, translation, and mitochondria. We propose that failure to sequester mRNA targets in *A1cf* mutant heterozygotes leads to functional differences among spermatids, thereby providing an opportunity for selection among haploid gametes. The study adds to the understanding of the gamete interaction at fertilization. Discovery that bias is evident with IVF provides a new venue for future explorations of preference among genetically distinct gametes at fertilization for *A1cf* and other genes that display significant departure of Mendelian inheritance.

## Introduction

The random nature of fertilization seemed so obvious that Mendel did not include it in his Laws, even though it is essential for understanding the inheritance of phenotypic variation. Mendel’s appreciation for the fundamental role of chance in sexual reproduction is evident in his use of binomial probabilities to convert frequencies of haploid gametes (*A* or *a*) into frequencies of diploid individuals (*AA, 2Aa, aa*)^1^. To ensure Mendelian inheritance, haploid gametes in each sex must be functionally equivalent^2^. The strong selective pressure to maintain equal transmission limits phenotypic variation during meiosis, among gametes, and at fertilization^3,4^. Cytoplasmic bridges promote phenotypic similarity by enabling transfer of RNAs, proteins and other cellular constituents among the four genetically unique products of each meiosis^5–7^. However, some gene products are found primarily in the gamete in which they originate. This restricted localization can lead to phenotypic variation among haploid gametes^8–11^, creating opportunities for preferential transmission, with several of these genes carrying signatures of positive natural selection^12^.

Some genetic variants violate the Law of Independent Segregation by preferentially transmitting alleles from heterozygotes to offspring^3,13–15^. Several kinds of distorted transmission have been reported. Meiotic drive results from preferential segregation of alleles through meiosis. Transmission ratio distortion results from differences in gamete numbers and activity. Deviations from Mendelian expectations can also result from selective embryo, neonatal, or juvenile loss. Distortion is usually a property of one sex and is independent of the genetic constitution of their mating partner^3,4,15^. Although generally rare, examples of transmission distortion have been reported in many species^3,13–15^.

**Figure 1:**
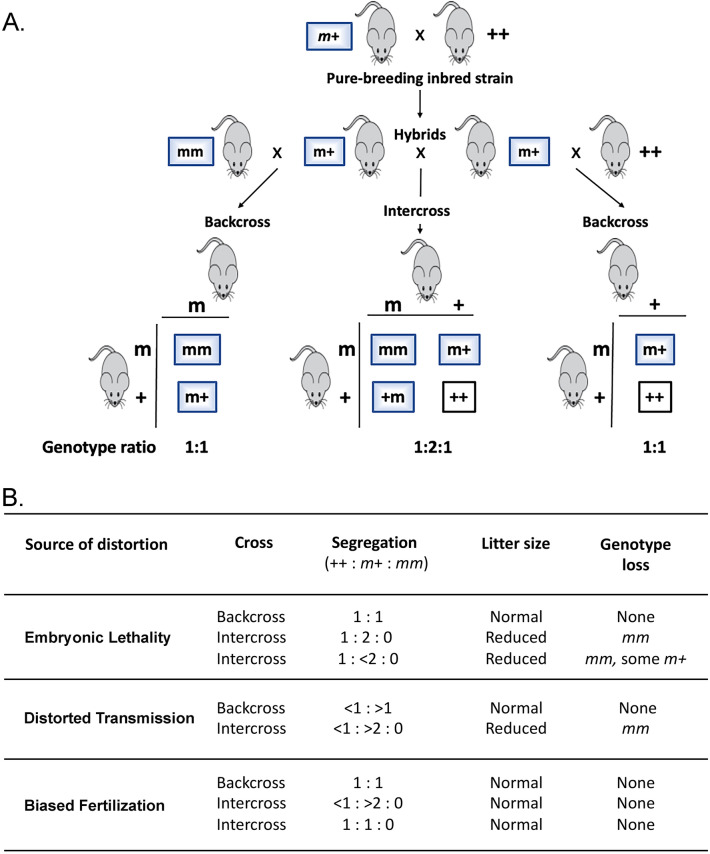
Mendelian segregation and deviations from expectations. (**A**) Mendelian segregation. ‘*m*’ and ‘+’ designate the mutant and wild-type allele, respectively. Intercrosses between inbred strains make F1 hybrids. Hybrids are then crossed to either parental strain for backcrosses or *inter se* for intercrosses. Genotype ratios are based on Mendelian expectations. (**B**) Distinct segregation ratios, litter size, and genotype loss depending on the basis for non-Mendelian segregation^66^. With embryonic lethality, mutant alleles that compromise viability of *mm* homozygotes can also result in loss of some *m*+ heterozygotes (partial dominance), which can further reduce litter size. Intercrosses can show an excess (< 1: > 2) or a deficiency (1:1) of *m*+ relative to ++.

Discovery that fertilization can depend on the genetic constitution of *both* oocyte and sperm highlights a largely unexplored dimension of haploid selection^16–18^. For example, among offspring of mouse crosses involving an engineered mutation in the *A1cf* gene (APOBEC1 complementation factor), a strong excess of heterozygotes was observed, as high as ~ 13 mutant heterozygotes found for every wild-type homozygote^19^, instead of the 2:1 ratio expected for Mendelian inheritance in intercrosses (Fig. 1A). At least eight other genes similarly affect fertilization, with some yielding an excess of heterozygotes and others a deficiency^18^. For these nine genes, mutant homozygotes are invariably absent.

Three hypotheses could explain departures from Mendelian expectations: Embryonic Lethality, Distorted Transmission, and Biased Fertilization^18^ (Fig. 1B). Normal litter sizes in intercrosses argue against embryo loss and normal segregation in backcrosses argues against sex-specific distorted transmission^18^ (see Fig. 1 for a description of the various crosses). Absence of mechanisms to compensate for embryo loss supports this argument. Instead, for these “bias genes”, genotype distortion without reduced litter size argues that fertilization can sometimes be genetically biased towards particular combinations of oocyte and sperm^18^ (Fig. 1B).

The basis for such biased fertilization is unknown. Especially, it is unclear whether bias results from interactions between gamete-specific factors such as cell-surface receptors and ligands^20–22^ and/or external factors acquired by sperm during their transit in the epididymis^23,24^ or by oocytes during their maturation in the oviduct^25,26^, genetic interactions between factors in eggs and sperm during completion of meiosis at fertilization^27,28^, or from somatic factors that facilitate fertilization in the oviduct^29–31^. Here, we used in vitro fertilization (IVF) to test for gamete- versus soma-specific control of biased fertilization. We also measured reproductive features to test alternative explanations for bias and mined available transcriptomes to identify targets of the A1CF RNA-binding protein that could drive haploid selection. We demonstrated that the fertilization bias specifically depends on the genetics of oocyte and sperm and propose that it originates from the functional differences between spermatids arising from sequestration of *A1cf* transcripts. Previously, evidence for bias was incidental to the primary focus of reported work. Here, we report the first study focused exclusively on the evidence for bias and tests for one of the proposed mechanisms.

## Results

### In vitro fertilization and in vivo validation support unusual *A1cf* segregation

To mitigate the influence of somatic effects from the parents on *A1cf* segregation, we obtained gametes, MII oocytes and cauda epididymal sperm, from *A1cf* ^*m+*^ parents and subjected them to in vitro fertilization (IVF). We examined a total of 332 IVF-produced embryos and a total of 1,198 mice resulting from various intercrosses (test) and backcrosses (control). To assess the relative contribution of gametes and soma to fertilization bias in vitro crosses were made with an appropriate combination of oocytes and sperm from *A1cf*^*m*+^ (*m*+) mutant heterozygotes and *A1cf*^++^ (++) wild-type homozygotes (Table 1, top). The ratio of *m*+ to ++ IVF-derived embryos (10:1) differed significantly from the expected 2:1 Mendelian ratio (χ^2^ = 110.46, *p* < 0.0001). By contrast, backcross IVF results were consistent with expectations. Embryos derived from these crosses progressed similarly through early developmental stages in vitro (Table 1, bottom). The significant departure from expectations shows that bias depends largely on factors intrinsic to gametes from *A1cf*^*m*+^ heterozygotes, or perhaps on a genetically determined predisposition of gametes to somatic influences taking place during epididymal sperm maturation and oviductal oocyte transport persisting after gametes are isolated for IVF tests.

**Table 1:** In vitro fertilization (IVF) and embryo culture for intercrosses and backcrosses.

IVF for intercrosses and backcrosses					
Cross	Embryo genotype			Genotype ratio	Sex ratio
	++	*m*+	*mm*	*m*+/++	F/M
Intercross	15	150	4	10.0	0.9
				*expect 2.0*	*expect 1.0*
Backcross, *m*+ female	14	15	na	1.1	1.6
				*expect 1.0*	*expect 1.0*
Backcross, *m*+ male	58	76	na	1.3	1.1
				*expect 1.0*	*expect 1.0*

Embryo development after IVF					
Cross	No. IVFs	No. oocytes inseminated	No. 2-cell embryos (%)	No. M/EB (%)	No. B/ExB (%)
Intercross	9	805	456 (57)	416 (91)	318 (70)
Backcross	16	972	494 (51)	444 (90)	324 (66)

For embryo development after IVF, the results for the reciprocal backcrosses were pooled. Percentage calculations: 2-cell embryos from inseminated oocytes; M/EB and B/ExB from 2-cell embryos.

M, morulae; EB, early blastocysts; B, blastocysts; ExB, expanded blastocysts.

**Figure 2:**
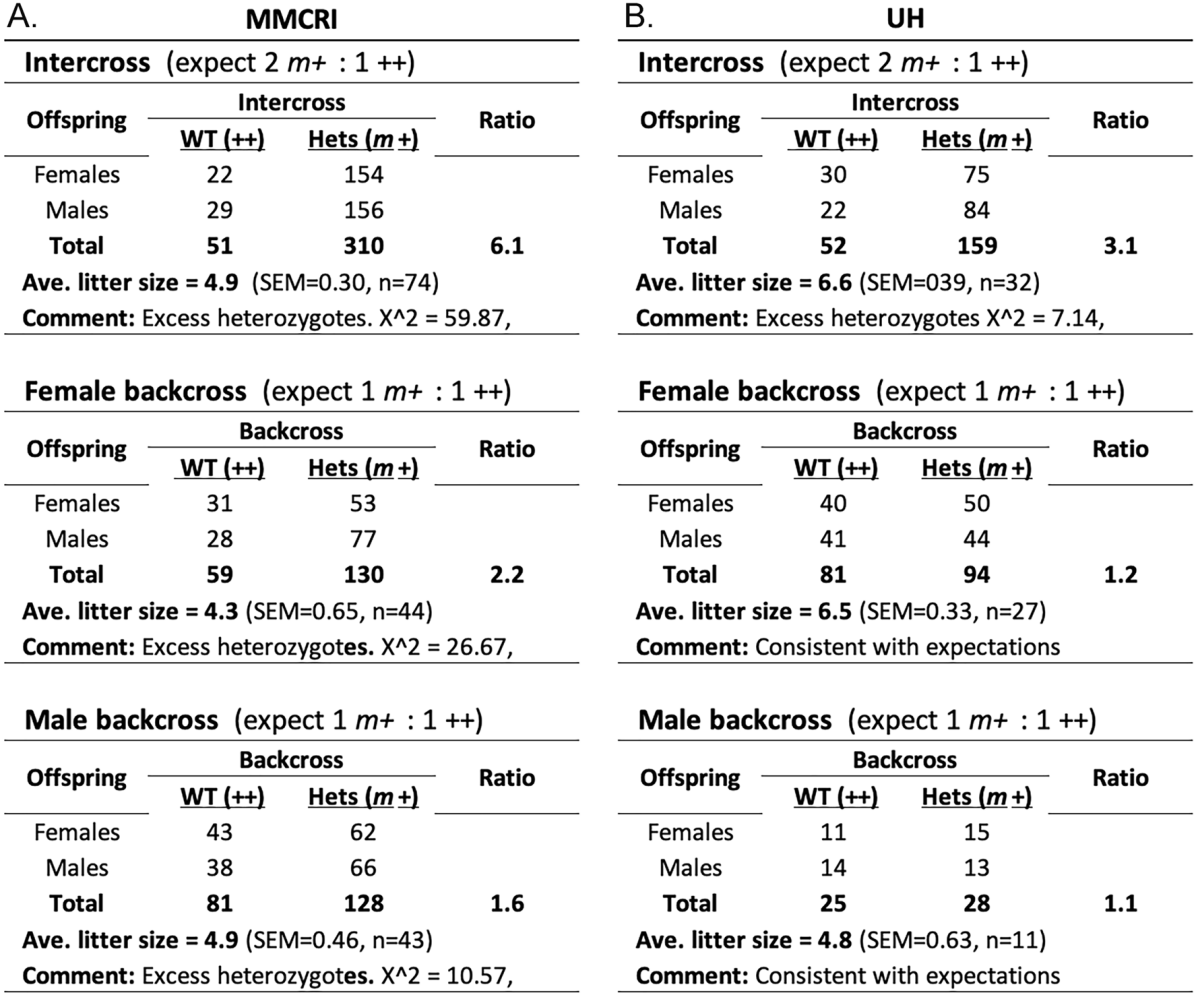
*A1cf* segregation in in vivo test and control crosses. *A1cf* genotype segregation is shown for two study locations: the Maine Medical Center Research Institute (MMCRI, panel **A**) and the University of Hawaii (UH, panel **B**). ++ refers to the wild-type genotype and *m*+ to the heterozygous genotype. Homozygotes were not found in either intercross. c^2^ goodness-of-fit tests are shown with point-wise *p*-values; results remain significant after correcting for testing multiple hypotheses. ‘Comment’ refers to test results for the fit between total observed genotype numbers with Mendelian expectations for wild-type and heterozygous mice. Sex ratios did not differ significantly from 1:1 expectation (not shown). SEM, standard error of the mean; n, total number of litters.

To verify bias in vivo, *A1cf *^*m*+^ heterozygotes were intercrossed, and in parallel *m*+ females and males were backcrossed to ++ homozygotes (Fig. 2). These breeding experiments were performed at two locations, the Maine Medical Center Research Institute (MMCRI) and the University of Hawaii (UH). We found 6.1 m+ for every ++ offspring (6:1) in the MMCRI intercross and 3.1 m+ for every ++ offspring (3:1) in the UH intercross, instead of the 2:1 ratio expected for intercrosses. The intercross results were significant when based either on Mendelian expectations or on the observed backcross results (Text S1). Backcrosses at MMCRI but not UH showed modest departures from expectations (Fig. 2). Strong bias in intercrosses has been reported in previous studies (Table S1). The somewhat variable magnitude of bias between UH and MMCRI may be explained by the differences in mouse source (mice derived recently from frozen embryos at UH versus a longstanding colony at MMCRI; see Materials and Methods), the possibility of microbiome differences in the reproductive tracts between sites^32–34^, undetected differences in local husbandry conditions, or sampling fluctuations. One or more of these mechanisms may also be responsible for the modest departure from expectations in MMCRI backcrosses, which was evident in both male and female backcrosses and thus resulted from alterations of both sperm and oocytes. An attractive possibility is a difference in diets between the two locations—an absence of soybean meal in the UH diet. Soybeans are a significant source of phytoestrogens that could modulate gametogenesis and fertility^35^, possibly serving as an environmental factor promoting biased *A1cf* segregation.

In the current study no *A1cf*^mm^ homozygotes were obtained in any of the mating crosses on the 129 genetic background. *A1cf*^mm^ homozygotes were observed before among embryos on mixed the 126-B6 genetic background^19^ and among offspring on the pure B6 genetic background with a mutation targeting a different side in the *A1cf* gene (MGI 1917115, mousephenotype.org) (Table S1). It is possible that B6 background carries at least one genetic variant that normalizes fertilization in *A1cf*^m+^ mice thereby enabling production of *A1cf*^mm^ homozygotes. Such genetic variants are extremely common^36^. It is also possible that type of mutation contributed to contrasting results between our study and that reported by mousephenotype.org.

### Analysis of three possible sources of distortion supports that Biased Fertilization is responsible for untypical *A1cf* transmission bias

We next evaluated the evidence for the three possible sources of bias: Embryonic Lethality, Distorted Transmission, and Biased Fertilization (Fig. 1B).

#### Embryonic lethality

Embryonic lethality does not readily account for *m*+ excess, absence of *mm* homozygotes, and deficiency of ++ among intercross offspring. Loss of *mm* should reduce litter size by 25% in intercrosses versus backcrosses where *mm* homozygotes do not occur. Litter size could be further reduced if some ++ or *m*+ mice are also lost (Fig. 1B). But in our study litter size did not differ substantially between intercrosses and backcrosses (Fig. 2). At MMCRI, the average litter size for intercrosses and combined backcrosses was 4.9 and 4.6 pups per litter, respectively, whereas at UH these were 6.6 and 6.0, respectively. By comparison, wild-type 129S1/SvImJ inbred mice have an average litter size of 4.9^37^. Finally, an average intercross litter sizes of ~ 8.4–10.0 at conception, which is roughly double the observed litter size for backcrosses in the MMCRI study and for the 129S1/SvImJ strain, would be needed to account for the observed loss of all *mm* and some ++ offspring (Text S2). Finally, when we evaluated extended MMCRI breeding data, we found that litter sizes were uniformly larger in intercrosses than in backcrosses and the 129S1/SvImJ strain, contrary to the expectation if some mm genotypes were lost postnatally (Table S2). From more than 1300 pups followed between birth and weaning, only six were lost, and all were in backcrosses rather than intercrosses. These lost pups occurred in each of the three types of backcrosses, suggesting loss was random rather than associated with a particular strain combination (Table S2). Together, these data show that it is unlikely that embryonic lethality contributes significantly to excess heterozygosity.

**Figure 3:**
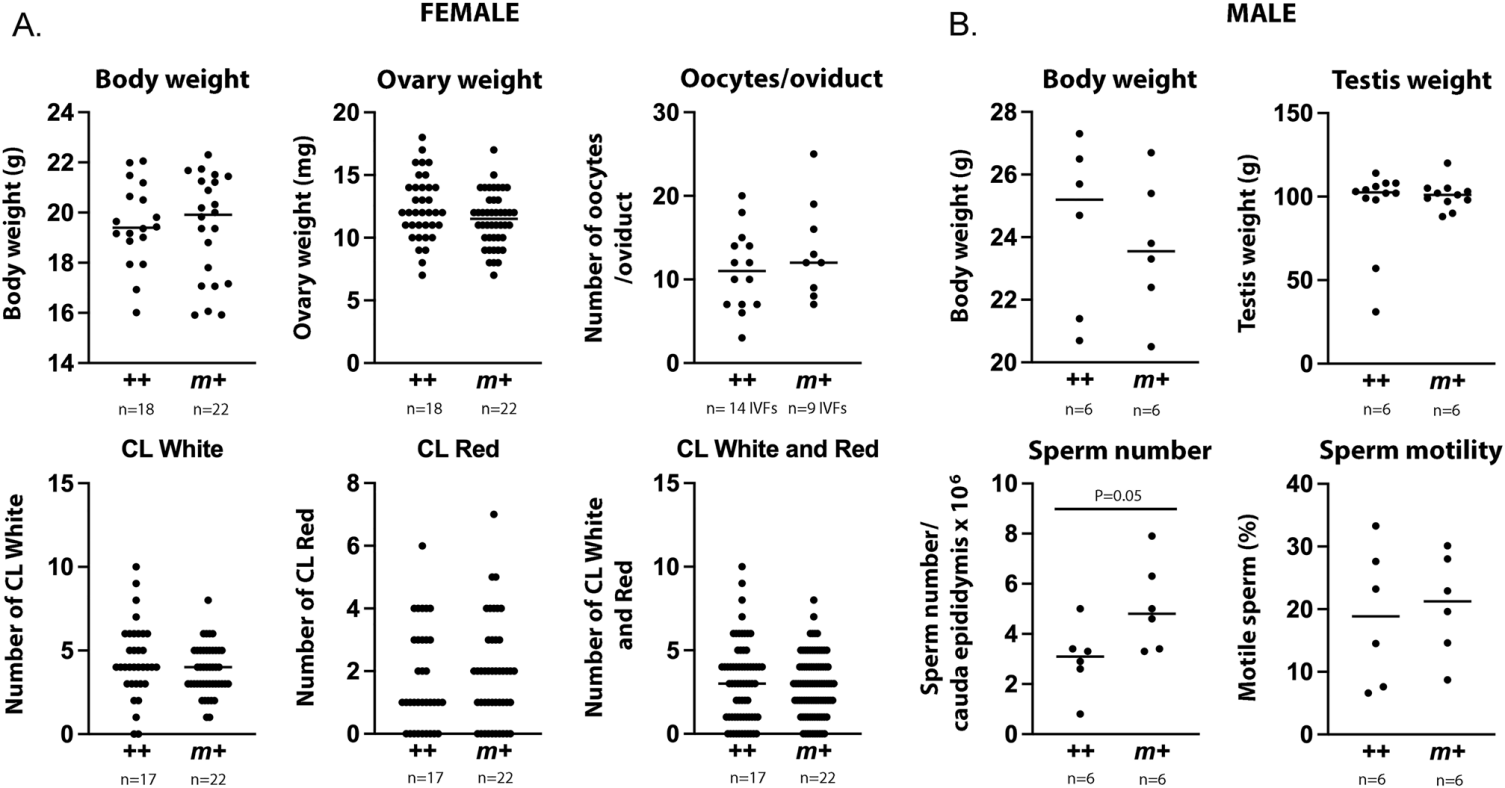
Reproductive features of *A1cf* mice. Female (**A**) and male (**B**) *A1cf m*+ and ++ mice were examined in regard to their reproductive features. The n shown under each graph represents a number of mice, unless stated otherwise. The number of oocytes per oviduct was assessed during IVF so each value was derived by dividing the total number of oocytes by the number of oviducts from which they were released in each IVF. The females used for IVF were subjected to hormonal stimulation. All mice were 8–10 weeks old. CL, corpora lutea; CL White, old CL that regressed to a fibrous scar; CL Red, fresh CL that was still hemorrhagic. Statistical analysis (*t*-test) revealed no significant differences between *A1cf m*+ and ++ mice for any measured parameter except sperm number.

Ovulation rate provides complementary evidence about embryo loss. The number of live-born offspring is closely related to the number of ovulated oocytes, which is largely determined by female genetics and is typically independent of the genetics of the mating partner^38^. Corpora lutea form where oocytes emerge from ovarian follicles at ovulation and serve as a direct measure of the number of ovulated oocytes^38^. At MMCRI, five intercross females examined at parturition had a total of 41 corpora lutea and 39 embryos. Resorbed intercross embryos were rare, only 1 of 101 embryos across 18 intercross litters, and were not found among embryos flushed from the oviduct at embryonic day 3.5^19^. Finally, in the UH study, none of the reproductive features, including the number of corpora lutea, differed significantly between *m*+ and ++ (Fig. 3). Thus, in neither study location did differences in corpora lutea numbers, resorptions, or other reproductive features account for differences in genotype numbers.

#### Distorted transmission

Transmission Ratio Distortion (TRD) is the preferential transmission of particular alleles from heterozygotes to offspring^2–4,13–15,39^. These exceptions to Mendelian inheritance are usually sex-specific and independent of the genetics of mating partners. If transmission distortion accounted for bias, similarly strong deviations from Mendelian expectations should be found in backcrosses as well as intercrosses (Fig. 1B).

Distortion observed in some backcrosses (Fig. 2) was too weak to account for the strong bias found in the intercrosses (Text S1).

#### Biased fertilization

For fertilization to cause bias, genotype distortion in embryos or offspring should not be linked to changes in litter size or genotype loss (Fig. 1B). All the data presented above (Table 1, Figs. 2, 3, Text S1–S2, Table S2) agree with these requirements.

Together, the data support that among the three possible sources of distortion, the evidence is consistent only with Biased Fertilization.

### Transcriptome data mining points to a possible mechanism for fertilization bias in* A1cf* transmission

**Figure 4:**
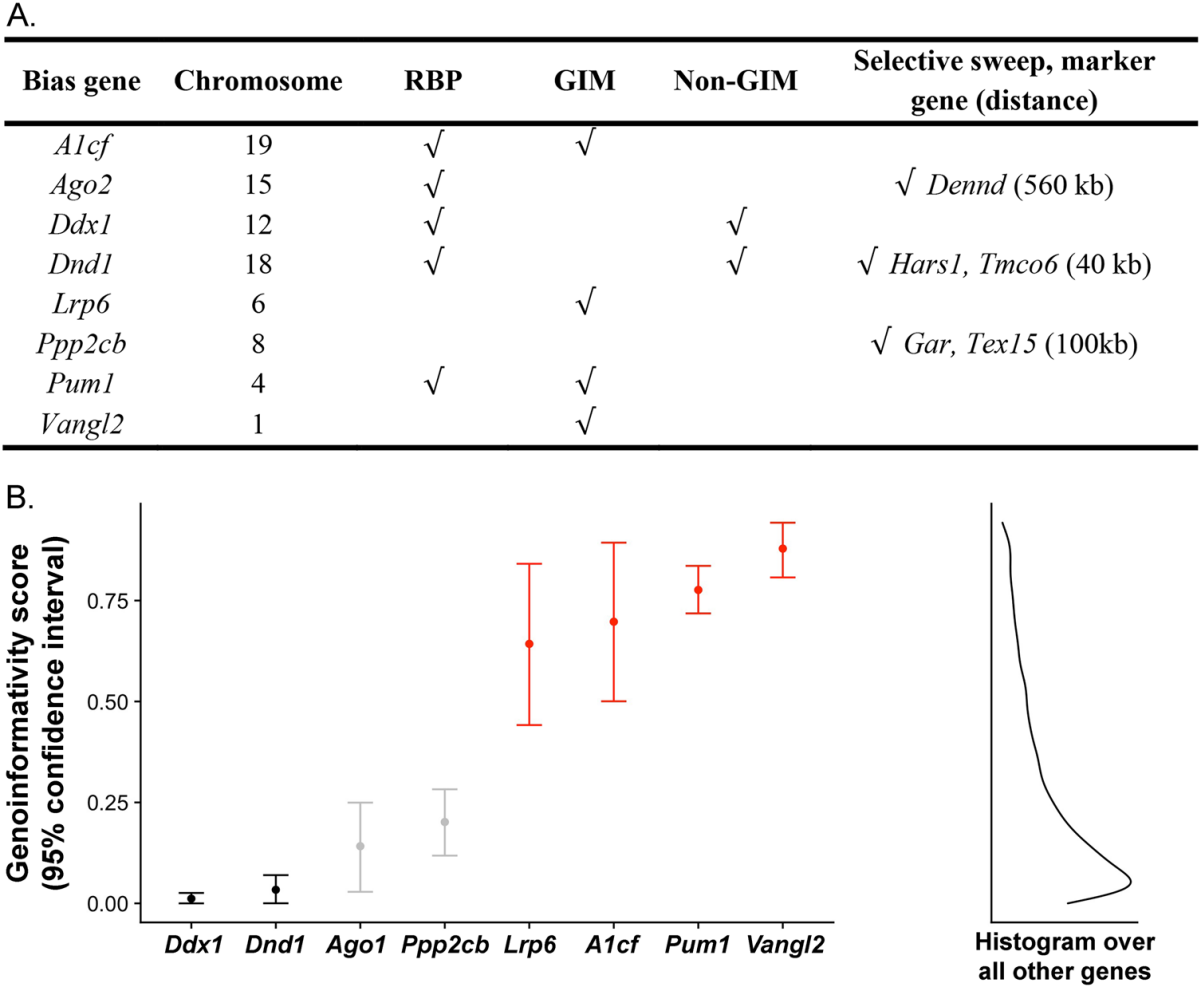
RNA-binding proteins and genoinformativity markers. (**A**) RNA-binding proteins (RBPs) and genoinformativity markers (GIMs). Source of information: Chromosome (informatics.jax.org), RBPs^18^, and GIMs, non-GIMs and sweeps^12^. The approximate distance (kb) of “bias” genes from the center of the sweep is provided in parentheses. The *Ppp2cb* transcript was detected but did not reach the threshold to determine whether it is a GIM or non-GIM^12^. The ninth ‘bias’ gene, apolipoprotein B transcript (*Apob*), which is not known to be an RBP, was not detected^12^. (**B**) Genoinformativity scores. Left: genoinformativity scores and 95% confidence intervals^12^ are displayed. Four are confident GIMs (red), two confident non-GIMs (black), and two “remaining genes” (gray). Right: the kernel density showing that most mouse genes had low genoinformativity scores.

What factors intrinsic to sperm might predispose them to bias? *A1cf* and four of the nine currently known “bias genes” encode RNA-binding proteins (RBPs) that control many aspects of RNA biology (Fig. 4A). A1CF is one of the mRNA targeting components of the APOBEC1 RNA editing complex^40^ and also controls splicing^41^. A1CF protein expression is high in mature oocytes at all ages examined as well as in testis, spermatozoa and epididymides, but declines with age in testes and epididymides^42^. AGO2 is part of the RISC complex that silences miRNAs, piRNAs and transposons^43^. DDX1 is an ATP-dependent RNA DEAD-box helicase involved in translation, splicing, and miRNA processing^44^. DND1 blocks miRNAs access to their mRNA targets thereby stabilizing target transcripts for translation^45^. PUM1 represses translation by binding 3′UTR’s of target transcripts, promoting miRNA access, and modulating mRNA stability^46^. The question is whether localizations of these RBPs or their targets is restricted to the gametes in which they originate, thereby creating opportunities for functional variation that could be the basis for haploid selection and biased fertilization.

To address this question, we examined previously published transcriptomes data^12^ for localized mRNA expression in spermatids from wild-type mice. This survey found more than 12,000 genes, including ‘genoinformative markers’ (GIMs) whose RNAs are enriched in the spermatid where they were transcribed as well as non-GIMs whose RNAs shuttle among connected spermatids. Interestingly, four of the eight “bias genes” are GIMs (*A1cf, Lrp6, Pum1, Vangl2*), two are non-GIMs (*Ddx1, Dnd1*), and two are ‘remaining’ genes that failed to reach the significance threshold (*Ago2, Ppp2cb*) (Fig. 4A,B). The GIM, non-GIM, and “remaining” genes distribution is consistent with that of all genes analyzed by reported by Bhutani^12^.

**Figure 5:**
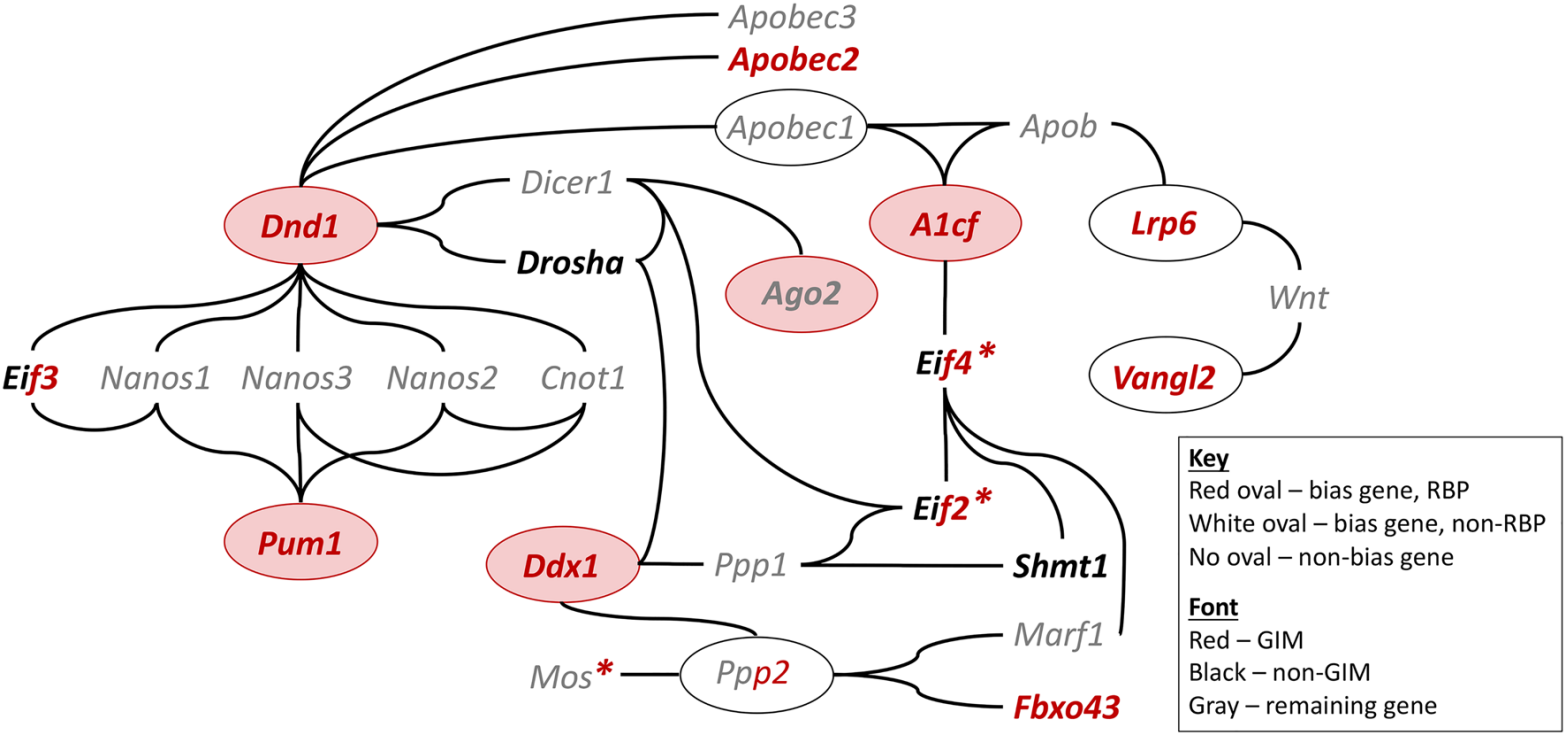
Network of interactions and functional dependencies. Genes were included if they show no more than two steps (nodes) from a bias gene. The *Eif* and *Ppp* gene families are shown in both red and black font because some family members are GIMs, others non-GIMs, and still others are ‘remaining genes’ or were not detected^12^. Red asterisks denote non-Mendelian segregation. See Table S5 and Fig. S3 for additional information.

As a first step to elaborate the network through which these genes bias fertilization, we asked whether mRNA targets of *A1cf* are also GIMs. A search for the A1CF-binding motif in mRNA 3’ UTRs of spermatid-expressed genes showed that these targets are enriched for GIMs compared to expression-matched, spermatid-expressed controls (*A1cf,* e-value < 0.005) (Fig. S1). Common functions among these *A1cf* targets include RNA biology, meiosis and fertilization (Tables S3, S4). Failure of A1CF to sequester these mRNA targets in *A1cf* mutant spermatids could compromise their localization and the resulting protein functions in both *m* and + spermatids in *m*+ heterozygous males. GIMs were also enriched among differentially spliced but not differentially expressed genes in mice with a liver-specific *A1cf* deficiency^41^ (χ^2^ = 6.40, 2 df, *p* < 0.05; Fig. S1). Similar analyses for enriched binding motifs and functions among mRNA targets of the other RBP GIM (*Pum1*) did not reveal notable trends (Fig. S2). Finally, several genes that interact directly with “bias genes” or that show altered function in mice with a mutation in a “bias gene” show non-Mendelian segregation (Table S5, Fig. S3). Collectively, these genes define a network of protein interactions and functional dependencies that coordinate mRNA stability, content, and translation control (Fig. 5). We propose that loss of sequestered transcripts in spermatids carrying the *A1cf* loss-of-function *m* allele leads to anomalous transcripts and protein products, and in turn to functional variation in both *m* and + spermatids in *m*+ heterozygotes. Similar effects might be found in mature *m*+ oocytes where *A1cf* is highly expressed^42^.

Because variants for the nine “bias genes” are either engineered or spontaneous mutations^18^, they have not been subject to natural selection. One could therefore argue that disrupting fundamental molecular features informs the mechanisms of fertilization in laboratory settings but does not necessarily guide understanding about the genetics of natural populations. We therefore asked whether “bias genes” have experienced positive selection, as expected for genetic variants that distort transmission. Several GIMs in mice and primates show evidence of selective sweeps in humans^12^, where the frequency of the selected genetic variant increases because of its fitness advantage^47^. Closely linked genetic variants hitchhike with the selected locus, showing correlated gene frequency changes depending on their recombination distance from the selected locus, and leaving a signature of reduced genetic variation. Interestingly, human orthologues of three “bias genes” (*Ago2, Dnd1, Ppp2cb*) are significantly enriched in selective sweeps (*p* < 0.0004; Fig. 4A), but whether “bias genes” drive these sweeps is uncertain.

## Discussion

Chance is central to sexual reproduction, from segregation and recombination to the union of gametes at fertilization. Although precise molecular mechanisms guide meiosis, recombination, and fertilization^48–50^, the unique combination of crossovers on each chromosome, chromosomes allocated to each gamete, and gametes that join at fertilization together make the genetic outcomes seemingly unpredictable. The statistical properties of Mendel’s Laws emerge from this mix of precision and chance. Exceptions such as transmission distortion drive preferential occurrence of alleles to the next generation, thereby reducing genetic variation and making outcomes more predictable.

### Genetics of gametes versus gene-environment interaction

A key question is whether the determinants of bias are intrinsic to gametes, or instead depend on gene-environment interactions. Factors in semen and from uterus, oviduct and ovarian follicles promote gamete viability, enhance chemotaxis and thermotaxis, and facilitate fertilization^29,31,51,52^. However, if these factors alone were sufficient for bias, gametes would be similarly affected regardless of their genetic content. Experimental in vivo approaches to this question face formidable challenges including the limited number of mature oocytes and sperm at fertilization, the transient nature of active gametes, and the ephemeral environment of the oviduct at fertilization. IVF obviates many of these challenges. The results reported here clearly show that bias depends on gamete genetics alone, or perhaps on a genetically-determined predisposition to environmental change that arises earlier during gametogenesis and persists after gametes are isolated for IVF tests.

### Functional consequences of bias genes

The function of bias genes can provide insights into molecular and cellular mechanisms that lead to the non-random union of gametes. During gametogenesis, some RNAs and proteins tend to remain within the spermatid in which they originate rather than translocate through cytoplasmic bridges that connect the four gametes resulting from each meiosis^5,7^. RBPs shuttle mRNAs to germ granules, such as chromatoid bodies, where their cargos can be stored, processed, or transported intra-cellularly and sometimes across these bridges^7,53^. Sequestered transcripts and proteins sometimes create functional differences among genetically distinct but otherwise phenotypically indistinguishable spermatids^8–11^. Any resulting phenotypic differences can be the basis for competition among gametes that sometimes leads to transmission distortion^8–11^. Interestingly, “bias genes” appear to be connected in a network of functional dependencies and protein interactions that coordinates mRNA stability, editing, and translation control with various germline processes (Fig. 5).

### Model

**Figure 6:**
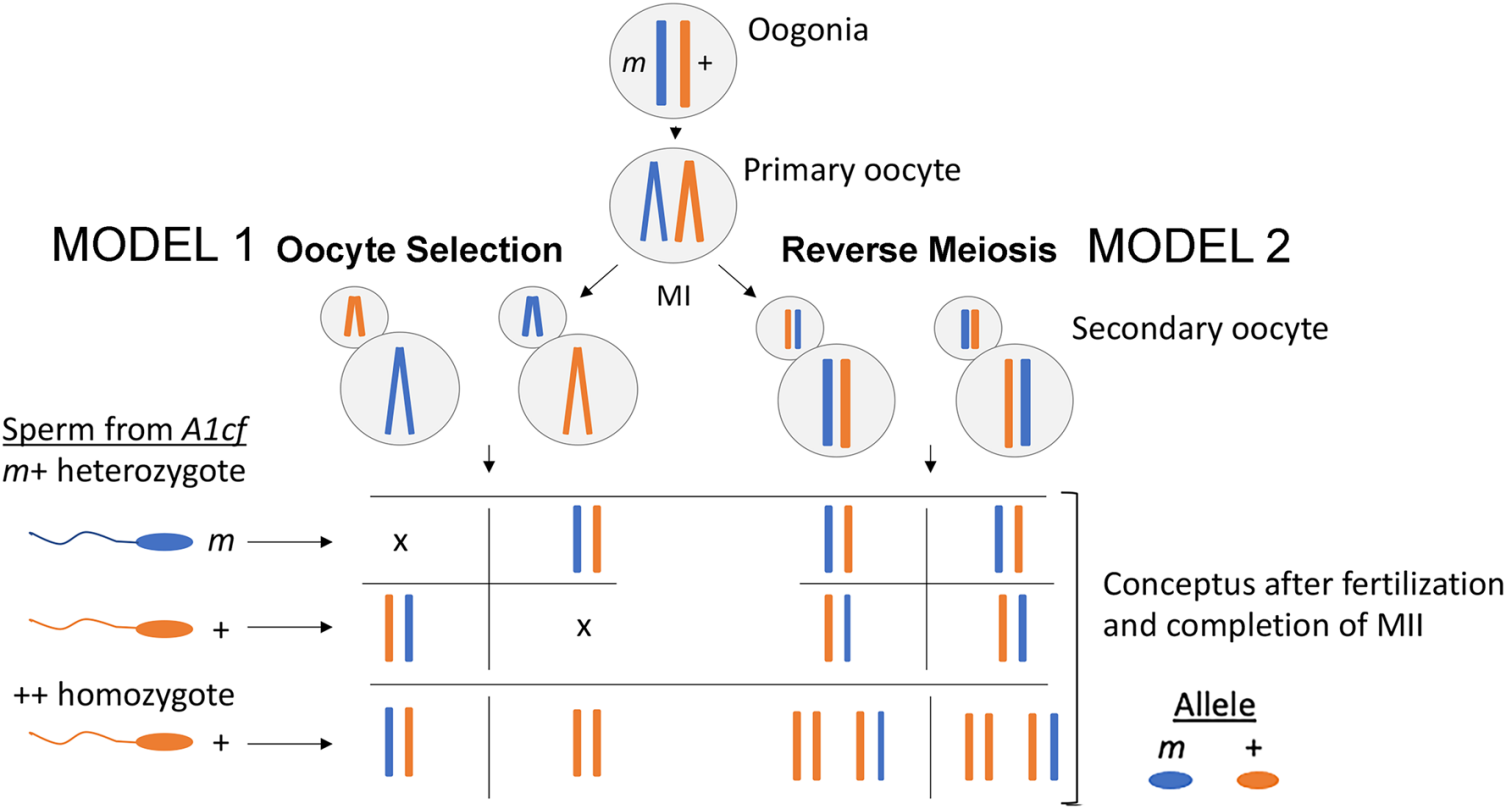
Two models for Biased Fertilization. Two models for putative effects of sperm and oocyte genotype on oocyte selection and meiosis are shown. According to the Oocyte Selection Model, sperm from *m*+ males preferentially or perhaps exclusively join with genetically heterologous ++ oocytes (or ++ sperm with *m*+ oocytes). By contrast, with the Reversed Meiosis Model, the genetics of fertilizing sperm determines which chromatid remains in the oocyte and which goes to the second polar body. In *A1cf* mice, the preference is to retain the chromatid with the opposite allele to that sperm brings so that heterozygous embryos predominate. Gametes with recombinant chromosomes are not shown.

Any model for biased fertilization must accommodate several observations. Sperm and oocytes carrying intrinsic functional differences in *A1cf* heterozygotes arrive at the site of fertilization in appropriate numbers and functionality, as normal segregation in the in vivo and in vitro backcrosses demonstrate. Most ovulated oocytes are fertilized, leaving little opportunity for selection among oocytes^54^. Finally, the window for bias in females is remarkably short, between the time when sperm trigger resumption of the second meiotic division (MII) and fusion of the female and male pronuclei^50^. Two models could account for biased fertilization (Fig. 6). The first involves Oocyte Selection where sperm bearing the *A1cf*+ allele selectively fertilize *mm* oocytes while those bearing the *m* allele fertilize ++ oocytes. Perhaps isoform variants within a species confer specificity to oocyte-sperm interactions as they do between species^20,55^. Alternatively, bias could result from a reversed order of meiotic divisions during oogenesis thereby creating an opportunity to direct chromatid segregation at MII and whereby retain a preferred chromatid in the oocyte, depending on the genetics of fertilizing sperm (Fig. 6, Reverse Meiosis). With the usual order of meiotic divisions where the reductional division precedes the equational division, preferential segregation has little impact given the genetic identity of sister chromatids at fertilization (unless recombination occurred at MI). However, if *A1cf* heterozygosity reverses the order of meiotic divisions during oogenesis, sperm genetics could lead to preferential retention of genetically distinct chromatids in ova at MII (Fig. 6). For example, sperm bearing the *A1cf m* allele could signal retention of the + but not the *m* allele in ova, leading to excess heterozygosity. There would be no possibility to detect selection if either parent was a wild-type homozygote. Several genes that regulate meiosis were found among *A1cf* targets in spermatids. If enrichment is genotype-specific, these transcripts might influence a completion of female meiosis post-fertilization. Reversed (inverted) meiosis has been reported in humans and mice^27,28^ and plant species with holocentric chromosomes^56^. In both models, preferential retention of recombinant chromosomes could augment or reduce the magnitude of bias^27,57^. Tests for these models are underway.

### Implications

In often elaborate ways diploid organisms choose mates; perhaps haploid gametes do the same. As Darwin proposed, mate choice depends on the perceived fitness of potential partners—fit mates tend to produce fit offspring. But gametes in such individuals are not equally fit. Although precise molecular mechanisms guide meiosis, recombination and fertilization, chance determines the combination of crossovers on each chromosome and chromosomes allocated to oocytes and sperm, making each gamete genetically unique. Sequestration of mRNAs and their protein products creates further functional diversity. As a result, fitness can vary among gametes even in fit individuals. Non-random joining of oocytes and sperm at fertilization could reflect haploid selection for fit combinations of gametes. Future in vitro as well as in vivo studies with *A1cf* and the other bias genes will help to clarify this interplay between chance and selection in gamete preference at fertilization.

## Materials and methods

### Mice

Work at both institutions used the same mutation (*129/Sv-A1cf*^*tm1Ddsn*^*/NaJ*) that was made and maintained on the 129S1/SvImJ inbred background (Jackson Laboratory, Strain #: 002448^19,42^. Mice for work at MMCRI were obtained from JHN’s breeder colony. Some of these mice were also donated to the Jackson Laboratory as a resource for the research community. Frozen embryos from the Jackson Laboratory resource were used to establish the UH breeder colony.

The *A1cf* (*A1cf*^*tm1Ddsn*^*,* MGI:3690371) mutants were made with a strain 129-derived ES cell line^19^ and then rederived on the 129S1/SvImJ inbred background, without outcrossing with C57BL6J or other strains^42^. Isogenicity greatly reduces the possibility of extraneous genetic effects. These mutant mice were provided to the Jackson Laboratory for preservation and distribution (Jackson Laboratory, Strain #: 027924). Mice at MMCRI were maintained on Envigo Teklad 2918 irradiated chow diet (Invigo RMS, Indianapolis, IN) provided ad libitum, at 70°F and with a 14L:10D schedule, with light-on at 7 am and light-off at 9 pm, Alpha Dry Plus bedding (Shepherd Specialty Paper, Watertown, TN), and in static cages (Allentown Inc., Allentown, NJ).

At UH, frozen embryos (*A1cf*^*tm1Ddsn*^, MGI:3690371) were acquired as a mixture of wild-type and heterozygous embryos (Jackson Laboratory, Strain #: 027924). Embryos were transferred into oviducts of CD-1 pseudo-pregnant recipients and resulted in heterozygous males and females that were used to establish a breeder colony. Mice were fed *ad libidum* with a standard diet (2020X Teklad Global Soy Protein-Free Extruded Rodent Diet, Harlan Laboratories, Indianapolis, IN) and were maintained with 7092 Teklad Corncob bedding (Envigo, Indianapolis, IN) in a temperature- and light-controlled room (22 °C, 14L:10D, with light-on at 7 am and light-off at 9 pm) in accordance with the guidelines of the Laboratory Animal Services at the University of Hawaii and guidelines presented in National Research Council’s Guide for Care and Use of Laboratory Animals published in 1996 by Institute for Laboratory Animal Research (ILAR) of the National Academy of Science (Bethesda, MD).

Experimental protocols for animal work were approved by the Maine Medical Center Research Institute Institutional Animal Care and Use Committee (protocols 1911 and 1912) or by the University of Hawaii Institutional Animal Care and Use Committee (protocol 06-010). The reporting in the manuscript follows the recommendations in the ARRIVE guidelines^58^.

### Media

Mineral oil was purchased from FUJIFILM Irvine Scientific (Santa Ana, CA); pregnant mares’ serum gonadotrophin (eCG) and human chorionic gonadotrophin (hCG) from ProSpec (East Brunswick, NJ). All other chemicals were obtained from Sigma Chemical Co. (St Louis, MO) unless otherwise stated. Medium HTF was used for IVF^59^, Hepes-buffered CZB (HEPES-CZB) for gamete handling^60,61^, and mKSOM^AA^ (KSOM medium containing NEAA and EAA^62^ for embryo culture. Both mKSOM^AA^ and HTF were maintained in an atmosphere of 5% CO_2_ in air and HEPES-CZB was maintained in air.

### In vitro fertilization and embryo culture

Sperm capacitation and IVF were performed as previously reported^63^. Briefly, oocytes were collected from females induced to superovulate with injections of 5 IU eCG and 5 IU hCG given 48 h apart. Epididymal sperm were collected by release from cauda epididymis directly into HTF medium and were capacitated for 1.5 h at 37 °C in a humidified atmosphere of 5% CO_2_. Gametes were co-incubated for 4 h, after which oocytes were washed with HEPES-CZB, followed by at least one wash with mKSOM^AA^ medium. Morphologically normal oocytes were selected for culture. Fertilized oocytes (oocytes with two well-developed pronuclei and extruded 2nd polar body) were cultured in 50 µL drops of mKSOM^AA^ medium pre-equilibrated overnight with humidified 5% CO_2_ in air. The number of fertilized 2-cell embryos was recorded after 24 h in culture. The 2-cell embryos were cultured in vitro to the blastocysts stage, with daily assessment of developmental progression.

### Mouse genotyping

#### MMCRI

PCR was used to amplify *A1cf* alleles as recommended by the Jackson Laboratory for some mice or with an in-house protocol for others. Genomic DNA was extracted from toe clippings with this use of the Hot Shot protocol (L. Liaw Lab, MMCRI; https://health.uconn.edu/mouse-genome-modification/protocols/hotshot-method-of-dna-preparation). Samples were exposed to a lysis buffer (25 mM NaOH, 0.2 mM EDTA, pH 12) at 95 °C for 45 min. before the extract was neutralized with 40 mM Tris HCl, pH 5.

Two PCR protocols were used: the Jackson Laboratory protocol and the in-house amplification protocol. The Jackson Laboratory protocol used three primers: a wild-type forward primer (24,997: GTC CAG CGC ACA GGA TAT AG), a mutant forward primer (oIMR7415: GCC AGA GGC CAC TTG TGT AG), and a common reverse primer (24,998: CAA AGG TCT AAC ATG GGC AGA). Amplifications were performed with positive, negative and blank controls. Because all samples were expected to yield a product, amplification control was not included. All reactions were performed in 30 μL, with overall primer concentrations of 0.33 μM for 24,997 and 24,998, and 0.40 μM for oIMR7415 in Taq Red Master Mix Kit (1.5 mM MgCl_2_) (Genesee Scientific, El Cajon, CA). The PCR cycling parameters began with an initial denaturation at 95 °C for 2 min followed with 28 cycles of denaturation (95 °C for 30 s), annealing (55 °C for 30 s), and elongation (72 °C for 30 s) followed by a final elongation step of 72 °C for 5 min. The in-house amplification protocol also used 3 primers: a common forward primer (Sse3: CAA ATC ACA AAT CCG GGG ATG), a wild-type reverse primer (ASse3a: CAA AGG TCT AAC ATG GGC AG) and a mutant reverse primer (ASse3b: ATAC TTC CAT TTG TCA CGT CC). Amplifications were performed with a positive, negative and blank controls. All reactions were performed in 30 μL with primer concentrations of 0.67 μM for Sse3 and 0.33 μM for both ASse3a and ASse3b in Taq Red Master Mix Kit (1.5 mM MgCl2) (Genesee Scientific, El Cajon, CA). The PCR cycling parameters began with an initial denaturation at 94 °C for 2 min followed with 35 cycles of denaturation (94 °C for 15 s), annealing (60 °C for 15 s), and elongation (72 °C for 20 s) followed with a final elongation step of 72 °C for 2 min. Amplified products (7–10 μL) were then electrophoresed on 1–1.5% agarose (VWR Chemicals, Solon, OH) gels with SYBR™ Green I (Invitrogen—Life Technologies Corporation, Eugene, OR) (0.5X concentration). The resulting products (Jackson Lab protocol: mutant 160 bp and wild-type 539 bp; in-house protocol: mutant 320 bp and wild-type 610 bp) were then visualized and photographed with a GE FUJIFILM LAS-4000 Luminescence Image Analyzer (FUJIFILM Corporation, Tokyo, Japan).

#### UH

PCR was used to amplify *A1cf* alleles as recommended by the Jackson Laboratory using the 24,997, oIMR7415 and 24,998 primers. Genomic DNA was obtained from ear clips and isolated using Qiagen DNeasy Tissue Kit (Qiagen, Valencia, CA). All amplifications were performed with positive, negative and blank controls. Because all samples were expected to yield a product, an amplification control was not included. The reactions were carried out in a volume of 15 µL, with 0.67 µM final concentration of each primer and using Go TaqGreen master mix (Promega, Madison, WI). The PCR conditions were as follows: initial denaturation at 95 °C for 2 min followed with 25 cycles with denaturation at 95 °C for 30 s, annealing at 55 °C for 30 s and extension at 72 °C for 30 s, followed with a final extension at 72 °C for 5 min. The PCR products (10 µL) were electrophoresed on 1.5% agarose (Lonza, Basel, Switzerland) gel containing 0.4 mg/ml ethidium bromide for 10 min at 100 V. Bands (539 bp *A1cf* wild-type, 160 bp *A1cf* mutant) were visualized with a UV transilluminator (Vilber Lourmat, Marnela-Valle’e, France) and photographed (Kodak, Los Angeles, CA).

### Embryo genotyping

Single blastocysts were sexed and genotyped for *A1cf* mutation simultaneously, using the same primers detecting the *A1cf* mutation that were used for mouse genotyping, duplexed with a primer pair amplifying a Y chromosome gene *Sry* (forward, MUTY3: GTG TCT CAA AGC CTG CTC TTC and reverse MUTYRP1: CAT GTA CTG CTA GCA GCT ATC). Single blastocysts were placed individually in the 0.2 mL PCR tubes in 1 µl volume of culture medium. The tubes were coded and frozen at − 20 °C until use. Four µL of GNTK buffer (50 mM KCl, 1.5 mM MgCl_2_, 10 mM Tris–HCl pH 8.5, 0.45% NP40, 0.45% Tween 20) and 1 µL of 2 mg/mL proteinase K were added to each tube. The tubes were heated at 65 °C for 10 min followed by 94 °C for 10 min, and cooled to 4 °C to achieve proteolysis and inactivation of proteinase K. The amplification reaction was carried out in a volume of 16.1 µL, with 0.62 µM final concentration of 24,997 and 24,998 *A1cf* primers, 0.74 µM of oIMR7415 *A1cf* primer, and 1.24 µM of combined (1:1) *Sry* primers, and using Go TaqGreen master mix. The PCR conditions were as follows: initial denaturation at 95 °C for 2 min followed with 28 cycles of denaturation at 95 °C for 30 s, annealing at 60 °C for 30 s, extension at 72 °C for 30 s and final extension at 72 °C for 5 min. The PCR products (10 µl) were electrophoresed on 1.5% agarose gel containing 0.4 mg/ml ethidium bromide for 10 min at 100 V. Bands (539 bp *A1cf* wild-type, 160 bp *A1cf* mutant, and 435 bp *Sry*) were visualized with a UV transilluminator and photographed.

### Analysis of corpora lutea

Ovaries were collected from 8 to 11 weeks old *A1cf*++ and *m*+ virgin females. Body weight and ovary weight were recorded, the ovaries photographed, and number of corpora lutea (CL) per ovary counted, with distinction between white (old CL that regressed to a fibrous scar) and red (fresh, still hemorrhagic CL).

### Sperm analyses

*A1cf*++ and m+ males were euthanized at 9–10 weeks of age. Body weight and testis weight were recorded. Sperm were obtained from cauda epididymides into HEPES-CZB and sperm number and percentage of motile sperm quantified.

### Prenatal loss

At term, females were sacrificed, and the number of live and dead embryos was recorded. Pups were not genotyped.

### Postnatal loss

The number of pups was counted at birth and at weaning, with any difference attributed to postnatal lethality. Pups were genotyped at weaning. Because the purpose here was to assess the extent of postnatal loss and whether such loss could account for absence of *mm* homozygotes, viability and genotyping results are reported separately.

### Outcome measures and statistics

The experiments were designed to compare observed and expected genotype distributions for intercrosses and backcrosses (Fig. 1A) as well as for embryos after IVF.

In the breeding studies, the following outcomes were measured: (1) litter size; (2) *m*+:++ ratio; and (3) sex ratio (the numbers of females and males). In the IVF study, the following outcomes were measured: (1) number of oocytes per oviduct; (2) the proportion of 2-cell embryos obtained from oocytes inseminated; (3) the proportion of morulae and blastocysts obtained from cultured 2-cell embryos; (4) the blastocyst *m*+:++ ratio; and (5) the blastocyst sex ratio. The additional measures included analyses of *m*+ and ++ *A1cf* females (body weight, ovary weight, corpora lutea scores) and *m*+ and ++ *A1cf* males (body weight, testis weight, sperm number, sperm motility).

Depending on the study, chi-square goodness-of-fit or chi-square contingency tests were used, both with 1 degree of freedom. Statistical significance was claimed for tests showing *p* < 0.05, both point-wise as well as after experiment-wide Bonferroni correction for multiple testing. The test score and its point-wise probability are provided; the results remain significant after accounting for testing six crosses. Data analysis and computations were done with Excel and GraphPad Prism.

#### Network analysis

Several databases were queried to identify candidate partners of that interact directly with “bias proteins” (protein–protein interactions), that show functional dependency in mutant mice, or that are no more than two nodes away from “bias proteins” in interaction networks. The evidence was derived from the primary literature or from various genomic databases (uniport.org, string-db.org). Given the incomplete nature of the surveys for interactions, and the lack of systematic studies across tissues, stages of development, environmental conditions, and genetic backgrounds, it is possible that some interactions have not yet been documented. In some cases, at least one member of a gene family was counted as a putative interactor with a bias protein. For example, DDX1 interacts with PPP1R8*,* but the literature is silent about interactions with PPP2CB (string-db.org)*.* Because PPP2CB contributes to control of meiosis in oogenesis^64^, we connect DDX1 with the PP2 family and mark the interaction as ‘provisional’. Similarly, A1CF interacts with at least one member of the eIF4 translation family but interactions have not been tested with other members of the family. Finally, only those interactions were included that provide evidence for interactions among the bias genes that nucleate the network. Segregation data were obtained from mousephenotype.org or the literature.

#### Motif enrichment

For each gene expressed (TPM > 10) in mouse spermatids in Bhutani et al.^12^, we retrieved the annotated 3′ UTR sequences for the highest-expressed Ensembl transcript. Using canonical IUPAC motifs (WUAAUUR for *A1cf* and UGUANAUA for *Pum1*), we searched for enrichment using AME from the MEME Suite^65^. Position weight matrix (PWM) was used to evaluate the match between candidate RNA targets of *A1cf* and *Pum1* motif sequences. We searched for enrichment in GIM’s against the control background of expression-matched spermatid-expressed controls (all 20 sets) as reported in Bhutani et al.^12^. As an additional control, we searched for enrichment in the first control set against the other 19 control sets and verified that the adjusted p-values for each motif were greater than 1. To classify genes as GIM’s, non-GIM’s or remaining genes, we selected cutoffs on mean genoinformativity, confidence in the genoinformativity estimate, and confidence in haplotype calling for that region of the chromosome, that would in aggregate lead to a false discovery rate of no more than 10%. See Bhutani et al.^12^ for details.

#### GO ontology enrichment analysis

Enrichment analysis of binding partners for *A1cf* and *Pum1* for gene ontology (GO) terms was performed using the goana function in the limma package using all genes as the denominator*.* Results were truncated at *p* < 0.01 for *A1cf* (n = 121) and *p* < 0.001 for *Pum1* (n = 56). The threshold was more stringent for *Pum1* than *A1cf* because the collective results were highly heterogeneous, with few themes beyond ‘transcription’. Descriptors for these prioritized GO terms were distilled into several higher-level summary terms, e.g. ‘RNA’ included functions such as splicing, RNA transport and polyadenylation, and ‘meiosis’ included recombination and centrosomes. ‘Other’ included heterogeneous functions. The GO term id’s and descriptors were updated with the corresponding GO hierarchies and gene lists (Mouse Genome Database, informatics.jax.org), especially in cases where the descriptor was general, e.g. metabolic process (GO:0008152). In some cases, the primary literature was also reviewed.

#### Selective sweep

Data were obtained from Bhutani et al.^12^. To calculate the probability of finding three of nine “bias genes” in selective sweeps, we assumed that genome-wide 100 sweeps involved a total of 50 Mb (average 500 kb per sweep) corresponding to a probability of 0.017 that any three “bias genes” occur in the total area.

## Supplementary Information

Supplementary Information.

## Data Availability

All data are available in the main text or the supplementary materials.
