# Supplementary material for: Genetically-biased fertilization in APOBEC1 complementation factor (A1cf) mutant mice
Source: Sci Rep. 2022 Aug 10;12:13599. doi: 10.1038/s41598-022-17948-9 (PMC9365768; doi:10.1038/s41598-022-17948-9)
Supplement: Supplementary file 1 — Supplementary Information. [file 41598_2022_17948_MOESM1_ESM.pdf]

Supplementary Information

Genetically-biased fertilization in APOBEC1 complementation factor (*A1cf*) mutant mice

Naoki Hirose[1], Genevieve Blanchet[1], Yasuhiro Yamauchi[1], Abigail C. Snow[2], Robin Friedman[3], Carmen Y. Khoo[4], Christine W. Lary[4], Monika A. Ward[1], and Joseph H. Nadeau[2]

[1] Institute for Biogenesis Research, John A. Burns School of Medicine, University of Hawaii at Manoa, Honolulu, Hawaii; [2] Center for Molecular Medicine, Maine Medical Center Research Institute, Portland, Maine; [3] Ohana Biosciences, Cambridge, Massachusetts; [4] Center for Outcomes Research, Maine Medical Center Research Institute, Portland, Maine

Supplementary Material includes:

Text S1. Does bias in backcrosses fully account for bias in intercrosses at MMCRI?

Text S2. Does loss of wild-type and homozygous mutant offspring account for excess heterozygosity in the intercrosses?

Table S1. Comparison of the present *in vivo* results with published studies.

Table S2. Extended breeding data for intercrosses and backcrosses – postnatal loss.

Table S3. Gene ontology (GO) analysis of mRNA targets – summary.

Table S4. Gene ontology (GO) analysis of *A1c*f and *Pum1* mRNA targets.

Table S5. Evidence for deviations for Mendelian segregation.

Fig. S1. Candidate target RNA in *A1cf* mutant mice.

Fig. S2. mRNA targets of *A1cf* and *Pum1* RNA-binding proteins.

Fig. S3: Gene families with evidence for GIMs or deviations from Mendelian segregation.

References:

Text S1. Does bias in backcrosses fully account for bias in intercrosses at MMCRI?

Observation
Genotype frequencies in backcrosses differed significantly from 1:1 expectations.

Parameters Observed allele frequencies for heterozygous females (*m*+) crossed to wild-type males and for heterozygous males (*m*+) crossed to wild-type females:

Observed numbers

	++	*m*+	total	ratio
Intercross	51	310	361	6.1
m+ female backcross	59	130	189	2.2
m+ male backcross	81	128	209	1.6
Total			759	

Sex-specific transmission frequency of m *and + alleles*

m+ female backcross: + = 0.31 (= 59/189) *m* = 0.69 (= 1 – 0.31)
m+ male backcross: + = 0.39 (= 81/209) *m* = 0.61 (= 1 – 0.39)

Calculations
Expected genotype frequencies in the intercross for *m*+ females x *m*+ males based on the observed transmission frequencies in the backcrosses and normalized to account for absence of *mm* homozygotes:

Genotype	Normalized frequency	Normalized number
++	0.20 (= 0.31 x 0.39/0.59)	72.2 (= 0.20 x 361)
m+	0.80 (= 1.00 – 0.20)	288.8 (= 0.80 x 361)
mm	not observed	not observed

Expected and observed genotype numbers

	++	*m*+	Ratio *m*+/++	
Additive bias	72.2	288.8	4.0	
Observed bias	51	310	6.1	$\chi^2 = 7.78$, 1 df, $P<0.006$.

Conclusion
The magnitude of bias in the MMCRI intercross is significantly greater than expected if the intercross result was simply the combined (additive) bias for heterozygous females and males as estimated in the reciprocal backcrosses.

Text S2. Does loss of wild-type and homozygous mutant offspring account for excess heterozygosity in the intercrosses?

Assumptions

We assume that the observed number of heterozygotes in intercrosses is correct, at least for this analysis. Abbreviations: 'ic' – intercross, 'bc' – backcross.

MMCRI measures (see also Fig. 2A)

	Genotypes				Litter	
Cross	++	*m*+	*mm*	total	Number	Average
intercross	51	310	0	361	74	4.9
pooled backcrosses	140	258	0	398	87	4.6

MMCRI – intercross calculations

Estimated number of ++ and *mm* homozygotes at conception:	155 (= 310/2)
Estimated number of ++ and *mm* lost:	104 ++ (= 155 – 51)
	155 *mm* (= 155 – 0)
Estimated ++ and *mm* lost:	259 (= 104 + 155)
Estimated intercross average number lost per litter:	3.5 (= 259/74)
Estimated intercross litter size at conception:	**8.4** (= 3.5 + 4.9)

UH measures (see also Fig. 2B)

	Genotypes				Litter	
Cross	++	*m*+	*mm*	total	Number	Average
intercross	52	159	0	211	32	6.6
pooled backcross	106	122	0	228	38	6.0

UH - calculations

Estimated number of ++ and *mm* homozygotes at conception:	79.5 (= 159/2)
Estimated number ++ and *mm* lost:	27.5 ++ (= 79.5 – 52)
	79.5 *mm* (= 79.5 – 0)
Estimated ++ and *mm* lost:	107 (= 27.5 + 79.5)
Estimated intercross average number lost per litter:	3.3 (= 107/32)
Estimated intercross litter size at conception:	**10.0** (= 3.3 + 6.7)

Conclusions

- Observed weighted average intercross and backcross litter sizes (pups per litter at birth) are remarkably similar at MMCRI, and also at UH.

	Intercrosses	Backcrosses
MMCRI	4.9	4.6
UH	6.6	6.0

- For selective embryo loss to account for genotype bias, litter size would need to be substantially larger in intercrosses than in backcrosses and larger than reported for the 129S1/SvImJ inbred strain that served as background for these studies. These estimates for intercrosses (8.4 pups per litter - MMCRI and 10.0 – UH) are much larger than observed for backcrosses 4.6 and 6.0 pups per litter at MMCRI and UH, respectively) or reported for the 129S1/SvImJ inbred strain (5.7 pups per litter, see Table S2; 4.9 pups per litter, see Table 1 in[1]).

- Together, evidence presented here and in the main text does not support the argument that embryo loss contributed to fertilization bias.

Table S1. Comparison of the present *in vivo* results with published studies.

Conclusion	Reference**/Cross	Sample size	Offspring genotype			Ratio m+/++	Mutant allele	Genetic background	Diets *
			++	m+	mm				
INTERCROSS									
**Strong heterozygote excess**	[2] / Line 1 in reference Table 2	131	13	164	0	**12.6**	$A1cf^{tm1Ddsn}$	129-B6 mix	SBM #2
**Strong heterozygote excess**	[2] / Line 2 in reference Table 2	177	14	117	0	**8.3**	$A1cf^{tm1Ddsn}$	129-B6 mix	SBM #2
**Strong heterozygote excess**	This study (MMCRI)	361	51	310	0	**6.1**	$A1cf^{tm1Ddsn}$	129S1/SvImJ	SBM #4
**Strong heterozygote excess**	[2] / Line 3 in reference Table 2	20	0	20	0	20 / 0	$A1cf^{tm1Ddsn}$	129-B6 mix	SBM #2
**Heterozygote excess**	[3]	304	75	229	0	**3.1**	$A1cf^{tm1Ddsn}$	129S1/SvImJ	SBM #2
**Heterozygote excess**	This study (UH)	211	52	159	0	**3.1**	$A1cf^{tm1Ddsn}$	129S1/SvImJ	SBM 0
Mendelian	MGI 1917115[#] / IMPC	57	16	29	12	1.8	$A1cf^{tm1b(EUCOMM)Hmgu}$	B6N	SBM #6
BACKCROSS									
**Heterozygote excess**	This study (MMCRI) / *m*+ female	189	59	130	nd	**2.2**	$A1cf^{tm1Ddsn}$	129S1/SvImJ	SBM #4
**Heterozygote excess**	This study (MMCRI) / *m*+ male	209	81	128	nd	**1.6**	$A1cf^{tm1Ddsn}$	129S1/SvImJ	SBM #4
Mendelian	[3] / *m*+ female	525	255	270	nd	1.1	$A1cf^{tm1Ddsn}$	129S1/SvImJ	SBM #2
Mendelian	[3] / *m*+ male	499	234	265	nd	1.1	$A1cf^{tm1Ddsn}$	129S1/SvImJ	SBM #2
Mendelian	This study (UH) / *m*+ female	175	81	94	nd	1.2	$A1cf^{tm1Ddsn}$	129S1/SvImJ	SBM 0
Mendelian	This study (UH) / *m*+ male	53	25	28	nd	1.1	$A1cf^{tm1Ddsn}$	129S1/SvImJ	SBM 0

[#] mousephenotype.org; * Diets: **SBM #2**: LabDiet 53WU PicoLab Rodent Diet 20 5053, https://www.labsupplytx.com/wp-content/uploads/2012/10/5053.pdf. **SBM #4**: Envigo Teklad Global 18% Protein Rodent Diet, (SBM #4), https://insights.envigo.com/hubfs/resources/data-sheets/2018-datasheet-0915.pdf. **SBM #6**: LabDiet JL Rat and Mouse/Auto 6F; 5K52 https://www.labdiet.com/cs/groups/lolweb/@labdiet/documents/web_content/mdrf/mdi4/~edisp/ducm04_028039.pdf.
SBM 0 Envigo Teklad Global Soy Protein-Free Extruded Rodent Diet 2020X, (SBM 0), protein 19, fat 7, fiber, https://insights.envigo.com/hubfs/resources/data-sheets/2020x-datasheet-0915.pdf. Significant departures from Mendelian expectations are shown in bold. Abbreviations: nd – not defined, SBM – soybean meal.

Table S2. Extended breeding data for intercrosses and backcrosses – postnatal lethality.

Cross		No. litters	Ave. litter size (at birth)	Number		
**Female parent**	**Male parent**			**Born**	**Weaned**	**Lost**
Inbred strain						
129 ++	129 ++	9	5.7	51	51	0
Intercrosses						
129 *m*+	129 *m*+	11	7.5	82	82	0
(B6x129)F1 *m*+	(B6x129)F1 *m*+	15	7.6	114	114	0
(B6x129)F1 *m*+	(AJx129)F1 *m*+	3	11.0	33	33	0
(B6x129)F1 *m*+	(AJx129)F1 ++	16	11.3	181	181	0
(AJx129)F1 *m*+	(B6x129)F1 *m*+	12	8.5	102	102	0
(AJx129)F1 *m*+	(B6x129)F1 ++	21	9.4	197	197	0
	**total**	**78**		**709**		**0**
Backcross, m+ female						
129 *m*+	129 ++	28	5.8	162	161	1
129 *m*+	A/J ++	14	6.4	90	89	1
129 *m*+	B6 ++	16	7.3	116	115	1
Backcross, m+ male						
129 ++	129 *m*+	20	4.1	81	79	2
A/J ++	129 *m*+	22	4.9	107	106	1
B6 ++	129 *m*+	7	7.1	50	50	0
	**total**	**107**		**606**		**6**

Table S3. Gene ontology (GO) analysis of *A1cf* targets – summary.

Summary GO term	No pathways <0.001	<0.01
RNA	1	22
Meiosis	6	21
Nucleus	4	16
Mitochondria	3	13
Fertilization	0	2
Other	0	47

Full list of genes identified by GO for both *A1cf* and *Pum* is provided as Table S4.

Table S4. Gene ontology (GO) analysis of *A1c*f and *Pum1* mRNA targets.

A1cf

GO term id	GO descriptor	No. genes in GO term	No. test set genes	p-value
GO:0044614	nuclear pore cytoplasmic filaments	4	2	7.274E-05
GO:0045132	meiotic chromosome segregation	90	4	0.00029
GO:0006996	organelle organization	3885	26	0.00046
GO:0006091	generation of precursor metabolites and energy	526	8	0.00051
GO:0000280	nuclear division	407	7	0.00056
GO:0008655	pyrimidine-containing compound salvage	11	2	0.00066
GO:0043097	pyrimidine nucleoside salvage	11	2	0.00066
GO:0007127	meiosis I	114	4	0.00070
GO:0071840	cellular component organization or biogenesis	6697	37	0.00077
GO:0044615	nuclear pore nuclear basket	12	2	0.00079
GO:0061982	meiosis I cell cycle process	119	4	0.00082
GO:0007131	reciprocal meiotic recombination	53	3	0.00086
GO:0035825	homologous recombination	54	3	0.00091
GO:0042405	nuclear inclusion body	13	2	0.00093
GO:0048285	organelle fission	449	7	0.00100
GO:0003723	RNA binding	1950	16	0.00101
GO:0060009	Sertoli cell development	14	2	0.00108
GO:0005622	intracellular	15073	64	0.00122
GO:0051299	centrosome separation	15	2	0.00124
GO:0043484	regulation of RNA splicing	135	4	0.00131
GO:0043174	nucleoside salvage	16	2	0.00142
GO:0098687	chromosomal region	349	6	0.00142
GO:0016043	cellular component organization	6512	35	0.00211
GO:0060008	Sertoli cell differentiation	20	2	0.00222
GO:0005385	zinc ion transmembrane transporter activity	20	2	0.00222
GO:0005737	cytoplasm	11625	53	0.00228
GO:0000966	RNA 5'-end processing	21	2	0.00245
GO:0071577	zinc ion transmembrane transport	22	2	0.00269
GO:0051537	2 iron, 2 sulfur cluster binding	22	2	0.00269
GO:0006807	nitrogen compound metabolic process	10812	50	0.00292
GO:0140096	catalytic activity, acting on a protein	2165	16	0.00300
GO:0140013	meiotic nuclear division	172	4	0.00317
GO:0008535	respiratory chain complex IV assembly	24	2	0.00320
GO:0072509	divalent inorganic cation transmembrane transporter activity	24	2	0.00320
GO:0044424	intracellular part	15063	63	0.00325
GO:0015980	energy derivation by oxidation of organic compounds	285	5	0.00329
GO:0051307	meiotic chromosome separation	25	2	0.00347
GO:0035459	cargo loading into vesicle	25	2	0.00347
GO:0040032	post-embryonic body morphogenesis	1	1	0.00351
GO:0003322	pancreatic A cell development	1	1	0.00351
GO:0021905	forebrain-midbrain boundary formation	1	1	0.00351
GO:0021917	somatic motor neuron fate commitment	1	1	0.00351
GO:0021918	regulation of transcription from RNA polymerase II promoter involved in somat	1	1	0.00351
GO:0051878	lateral element assembly	1	1	0.00351
GO:0090616	mitochondrial mRNA 3'-end processing	1	1	0.00351
GO:0097222	mitochondrial mRNA polyadenylation	1	1	0.00351
GO:0051308	male meiosis chromosome separation	1	1	0.00351
GO:0042774	plasma membrane ATP synthesis coupled electron transport	1	1	0.00351
GO:0000802	transverse filament	1	1	0.00351
GO:0004490	methylglutaconyl-CoA hydratase activity	1	1	0.00351
GO:0050011	itaconyl-CoA hydratase activity	1	1	0.00351
GO:0033989	3alpha,7alpha,12alpha-trihydroxy-5beta-cholest-24-enoyl-CoA hydratase acti	1	1	0.00351
GO:0044594	17-beta-hydroxysteroid dehydrogenase (NAD+) activity	1	1	0.00351
GO:0004446	inositol-hexakisphosphate phosphatase activity	1	1	0.00351
GO:0030351	inositol-1,3,4,5,6-pentakisphosphate 3-phosphatase activity	1	1	0.00351
GO:0034417	bisphosphoglycerate 3-phosphatase activity	1	1	0.00351
GO:0052826	inositol hexakisphosphate 2-phosphatase activity	1	1	0.00351
GO:0052827	inositol pentakisphosphate phosphatase activity	1	1	0.00351
GO:0018025	calmodulin-lysine N-methyltransferase activity	1	1	0.00351
GO:0004654	polyribonucleotide nucleotidyltransferase activity	1	1	0.00351
GO:0070971	endoplasmic reticulum exit site	26	2	0.00375

A1cf

GO term id	GO descriptor	No. genes in GO term	No. test set genes	p-value
GO:0003824	catalytic activity	5710	31	0.00400
GO:0071514	genetic imprinting	27	2	0.00404
GO:0006829	zinc ion transport	27	2	0.00404
GO:1903046	meiotic cell cycle process	188	4	0.00435
GO:0043226	organelle	14144	60	0.00443
GO:0044238	primary metabolic process	11287	51	0.00449
GO:0044237	cellular metabolic process	11301	51	0.00464
GO:0046134	pyrimidine nucleoside biosynthetic process	29	2	0.00465
GO:0045333	cellular respiration	193	4	0.00477
GO:0050657	nucleic acid transport	193	4	0.00477
GO:0050658	RNA transport	193	4	0.00477
GO:0000775	chromosome, centromeric region	193	4	0.00477
GO:0051236	establishment of RNA localization	196	4	0.00504
GO:0000794	condensed nuclear chromosome	99	3	0.00513
GO:1903311	regulation of mRNA metabolic process	324	5	0.00565
GO:0072521	purine-containing compound metabolic process	613	7	0.00569
GO:0010464	regulation of mesenchymal cell proliferation	33	2	0.00599
GO:0009124	nucleoside monophosphate biosynthetic process	208	4	0.00620
GO:0043094	cellular metabolic compound salvage	34	2	0.00635
GO:0017004	cytochrome complex assembly	34	2	0.00635
GO:0006409	tRNA export from nucleus	34	2	0.00635
GO:0071431	tRNA-containing ribonucleoprotein complex export from nucleus	34	2	0.00635
GO:0009062	fatty acid catabolic process	108	3	0.00653
GO:0036111	very long-chain fatty-acyl-CoA metabolic process	2	1	0.00702
GO:0045720	negative regulation of integrin biosynthetic process	2	1	0.00702
GO:0035407	histone H3-T11 phosphorylation	2	1	0.00702
GO:2000828	regulation of parathyroid hormone secretion	2	1	0.00702
GO:2000790	regulation of mesenchymal cell proliferation involved in lung development	2	1	0.00702
GO:2000791	negative regulation of mesenchymal cell proliferation involved in lung development	2	1	0.00702
GO:0000962	positive regulation of mitochondrial RNA catabolic process	2	1	0.00702
GO:0046108	uridine metabolic process	2	1	0.00702
GO:0090615	mitochondrial mRNA processing	2	1	0.00702
GO:2000584	negative regulation of platelet-derived growth factor receptor-alpha signaling pathway	2	1	0.00702
GO:0032472	Golgi calcium ion transport	2	1	0.00702
GO:2000627	positive regulation of miRNA catabolic process	2	1	0.00702
GO:0099631	postsynaptic endocytic zone cytoplasmic component	2	1	0.00702
GO:1990723	cytoplasmic periphery of the nuclear pore complex	2	1	0.00702
GO:0045025	mitochondrial degradosome	2	1	0.00702
GO:0035402	histone kinase activity (H3-T11 specific)	2	1	0.00702
GO:0004137	deoxycytidine kinase activity	2	1	0.00702
GO:0051717	inositol-1,3,4,5-tetrakisphosphate 3-phosphatase activity	2	1	0.00702
GO:0000150	recombinase activity	2	1	0.00702
GO:0004850	uridine phosphorylase activity	2	1	0.00702
GO:0034416	bisphosphoglycerate phosphatase activity	2		0.00702
GO:0006406	mRNA export from nucleus	111	3	0.00704
GO:0071427	mRNA-containing ribonucleoprotein complex export from nucleus	111	3	0.00704
GO:0071704	organic substance metabolic process	12093	53	0.00710
GO:0051031	tRNA transport	36	2	0.00710
GO:0046915	transition metal ion transmembrane transporter activity	36	2	0.00710
GO:0044444	cytoplasmic part	9735	45	0.00741
GO:0034655	nucleobase-containing compound catabolic process	648	7	0.00764
GO:0019637	organophosphate metabolic process	1177	10	0.00787
GO:0097064	ncRNA export from nucleus	38	2	0.00789
GO:0000793	condensed chromosome	223	4	0.00789
GO:0034404	nucleobase-containing small molecule biosynthetic process	226	4	0.00826
GO:1901363	heterocyclic compound binding	6241	32	0.00841
GO:0022412	cellular process involved in reproduction in multicellular organism	357	5	0.00843
GO:1901564	organonitrogen compound metabolic process	7040	35	0.00866
GO:0006403	RNA localization	230	4	0.00878
GO:0008152	metabolic process	12552	54	0.00994

Pum1

GO term id	GO descriptor	No. genes in GO term	No. test set genes	p-value
GO:0140096	catalytic activity, acting on a protein	2165	24	9.374E-06
GO:0043484	regulation of RNA splicing	135	6	2.548E-05
GO:0048468	cell development	2183	23	3.445E-05
GO:0022008	neurogenesis	1623	19	4.817E-05
GO:1990841	promoter-specific chromatin binding	48	4	5.352E-05
GO:0048583	regulation of response to stimulus	4342	35	5.654E-05
GO:0006464	cellular protein modification process	4160	34	5.794E-05
GO:0036211	protein modification process	4160	34	5.794E-05
GO:0043412	macromolecule modification	4373	35	6.591E-05
GO:0048699	generation of neurons	1524	18	7.066E-05
GO:0001102	RNA polymerase II activating transcription factor binding	53	4	7.923E-05
GO:0044451	nucleoplasm part	1126	15	8.323E-05
GO:0005515	protein binding	11807	68	8.852E-05
GO:0005654	nucleoplasm	3512	30	8.861E-05
GO:0005694	chromosome	1075	14	0.00019
GO:0050767	regulation of neurogenesis	822	12	0.00020
GO:0050794	regulation of cellular process	11022	64	0.00021
GO:0030182	neuron differentiation	1365	16	0.00021
GO:0007610	behavior	594	10	0.00023
GO:0031175	neuron projection development	978	13	0.00027
GO:0005488	binding	15267	79	0.00030
GO:0008283	cell proliferation	2024	20	0.00030
GO:0030193	regulation of blood coagulation	79	4	0.00037
GO:0032762	mast cell cytokine production	7	2	0.00038
GO:0044260	cellular macromolecule metabolic process	8352	52	0.00039
GO:1900046	regulation of hemostasis	80	4	0.00039
GO:0051252	regulation of RNA metabolic process	3822	30	0.00041
GO:0050818	regulation of coagulation	84	4	0.00047
GO:0033613	activating transcription factor binding	85	4	0.00049
GO:0070847	core mediator complex	8	2	0.00050
GO:0048518	positive regulation of biological process	6254	42	0.00051
GO:0008134	transcription factor binding	661	10	0.00054
GO:0001085	RNA polymerase II transcription factor binding	155	5	0.00055
GO:0003682	chromatin binding	546	9	0.00056
GO:0120036	plasma membrane bounded cell projection organization	1497	16	0.00058
GO:0051960	regulation of nervous system development	927	12	0.00059
GO:0006366	transcription by RNA polymerase II	2818	24	0.00061
GO:0002327	immature B cell differentiation	9	2	0.00064
GO:0048522	positive regulation of cellular process	5490	38	0.00065
GO:0048731	system development	4918	35	0.00073
GO:0002244	hematopoietic progenitor cell differentiation	166	5	0.00075
GO:0006357	regulation of transcription by RNA polymerase II	2683	23	0.00075
GO:0060284	regulation of cell development	953	12	0.00076
GO:0030030	cell projection organization	1534	16	0.00076
GO:0017145	stem cell division	42	3	0.00078
GO:0051302	regulation of cell division	168	5	0.00079
GO:0009605	response to external stimulus	2520	22	0.00079
GO:0001221	transcription cofactor binding	43	3	0.00083
GO:0048666	neuron development	1110	13	0.00088
GO:0032502	developmental process	6411	42	0.00090
GO:0007399	nervous system development	2375	21	0.00091
GO:0043170	macromolecule metabolic process	10507	60	0.00095
GO:0140014	mitotic nuclear division	264	6	0.00097
GO:0043619	regulation of transcription from RNA polymerase II promoter in response to oxidative stress	11	2	0.00098
GO:0097493	structural molecule activity conferring elasticity	11	2	0.00098
GO:0005667	transcription factor complex	365	7	0.00099

Table S5. Evidence for deviations for Mendelian segregation.

Gene	GIM	Sex effect	Genotype numbers (++, *m*+, *mm*)	Ratio: ++:*m*+:*mm*	Test score	Source*	Comment
Eif2b4	non-GIM	combined	124, 165, 0	1 : 1.3	11.95, <0.0006	MGI: 95300	deficiency of *m*+
Eif4e	GIM	combined	29, 29, 0	1 : 1	7.31, <0.007	MGI: 95305	deficiency of *m*+
Eif4h	remaining	combined	103, 58, 0	1 : 0.6	67.91, <0.0001	MGI: 1341822	deficiency of *m*+
Fuz	remaining	male	32, 32, 0	1 : 1	8.06, <0.005	MGI: 1917550	deficiency of *m*+
Hook3	GIM	sex differences	30, 34, 11	1 : 1.3 : 0.4	10.28, <0.006	MGI: 2443554	sex-specific deficiency of *m*+ and *mm*
Mos	not detected	male	29, 25, 12	1 : 0.9	12.64, <0.002	MGI: 97052	sex-specific deficiency of *m*+
Odc1	not detected	female	1, 22, 0	1 : 22	8.76, <0.004	MGI: 97402	sex-specific excess of *m*+
Pik3c2a	GIM	combined	48, 56, 0	1 : 1.1	7.65, <0.006	MGI: 1203729	deficiency of *m*+
Ppp2cb	not detected	male	31, 28, 5	1 : 0.9	9.73, <0.002	MGI: 1321161	sex-specific deficiency of *m*+

* mousephenotype.org. Genes listed in Fig. S2 were surveyed to determine whether mice with a mutation in these genes show normal segregation and viability (mousephenotype.org). The genome survey is incomplete because some genes have not yet been mutated or mice phenotyped (mousephenotype.org). "Ratio" refers to +/+ and *m*/+ offspring of *m*/+ x *m*/+ intercrosses. Sex-specific effects refer to offspring. Chi-square goodness-of-fit tests for fit to Mendelian expectations (1 ++ : 2 m+) were used and point-wise p-values are reported. Absence of litter size in these reports precludes testing alternative explanations for bias (see Fig. 1B). "Combined" are pooled data for females and males. See Fig. S2 for additional information about these genes.

A.

Differentially spliced genes

Gene	Status		
Atp13a3	GIM		
Bace1	GIM		
Cdc27			remaining
Cd47	GIM		
Ears2			remaining
Fuz			remaining
Hook3	GIM		
Ifi27	GIM		
Kras	GIM		
Lrp6	GIM		
Map4k4	GIM		
Mapk15	GIM		
Mta1	GIM		
Myo18a	GIM		
Neb	GIM		
Nipsnap2			remaining
Numb			remaining
Pacsin2	GIM		
Pacsin3		non-GIM	
Papss2	GIM		
Picalm	GIM		
Pik3c2a	GIM		
Pitpnm1	GIM		
	**GIM**	**non-GIM**	**remaining**
**Percent total** (n)	**59.1** (26)	**13.6 (6)**	**27.3** (12)

Differentially spliced genes

Gene	Status		
Pitpnm2	GIM		
Plekha6	GIM		
Ppip5k2		non-GIM	
Pqlc1	GIM		
Ptcd3		non-GIM	
Rnf40			remaining
Rsrp1	GIM		
Scarb1	GIM		
Sec31a		non-GIM	
Sidt2			remaining
Sin3b			remaining
Slc7a2	GIM		
Slc39a14			remaining
Stx2	GIM		
Tmpo		non-GIM	
Ubxn1			remaining
Vps13b	GIM		
Wbp1			remaining
Zfp6			remaining
Zfp276	GIM		
Zkscan17		non-GIM	

B.

Differentially expressed genes

Gene	Status		
Abhd14b		non-GIM	
Acot11	GIM		
Adgrv1	GIM		
Capn10			remaining
Ceacam1		non-GIM	
Ces4a	GIM		
Fam234b		non-GIM	
Fgfr1		non-GIM	
Hmgcl			remaining
Hsd17b11	GIM		
Ifi27	GIM		
Il1rap		non-GIM	
Irf7			remaining
Lgals1	GIM		
Nipsnap2			remaining
Perp			remaining
Pex5	GIM		
Rgn	GIM		
Phkb	GIM		
Sugct	GIM		
Ttpa	GIM		
Tuba8			remaining
	**GIM**	**non-GIM**	**remaining**
**Percent total** (n)	**50.0** (11)	**22.7** (5)	**27.3** (6)

Fig. S1. Transcripts found in both *A1cf*-deficient liver as well as in spermatids from wild-type mice. Data from Nikolaou et al.[4] and Bhutani et al.[5] were surveyed to identify transcripts that were differentially spliced (**A**) or differentially expressed (**B**) in mice with liver-specific depletion of *A1cf* as well detected in spermatids from wild-type mice. Differential splicing and expression were reported in Nikolaou et al.[4]; expression in haploid spermatids was reported in Bhutani et al.[5]. GIMs were enriched among differentially spliced genes (($\chi^2 = 6.40$, 2 df, $p<0.05$) but were not significantly enriched for differentially expressed genes (not shown).

A1cf		A1cf		Pum1		Pum1	
Gene	PWM score	Gene	PWM score	Gene	PWM score	Gene	PWM score
Zmym1	37.18	Abhd3	5.96	Mrpl22	105.96	Klb	26.77
4930470P17Rik	32.66	Mark3	5.96	Fbxl13	98.93	Rras	26.53
Mrps28	23.37	Pdhb	5.89	Kif20b	92.89	Cd9	26.11
Catsper2	23.03	Arid4a	5.88	Ghrh	87.22	Cep192	25.76
Gm3409	19.64	Clk4	5.81	Txndc11	81.26	Gm4884	25.69
Gm3404	19.64	Ranbp2	5.80	Ilkap	78.00	Adgrb3	25.33
Abcg2	17.14	Speer4d	5.69	Gtf2b	64.62	Bcar1	25.22
Fdx1	15.98	Nup98	5.68	Dgcr2	64.01	Lmo1	25.04
Figla	15.56	4930572O03Rik	5.66	Tpte	59.82	Polr2a	24.91
Phf14	15.37	Hsd17b4	5.59	Rnf220	54.76	Mycbp2	24.91
Dnah7b	15.25	Utp20	5.50	Togaram2	53.37	Ttn	24.47
Dnah7c	15.25	Gm21083	5.50	Aspm	52.44	Agtr1a	24.33
Fam227b	14.18	Parp8	5.45	Mettl25	49.59	Clk4	23.97
Nbas	14.15	Lipo2	5.35	Gm5114	48.99	Gm5592	23.95
Tmeff2	14.12	Gm10354	5.24	Pcca	47.78	Brd8dc	23.82
Fkbp7	12.64	Slc39a12	5.23	Med31	47.48	Gak	23.30
Magoh	12.57	Snrpn	5.17	Cntn6	47.28	Map4k3	22.89
1700067P10Rik	12.04	Eme1	5.12	Cep44	45.46	Mia2	22.18
Pnpt1	11.77	Sycp1	5.12	Prss36	43.85	Polr3a	21.87
Prss36	11.74	Slc30a6	5.10	Fbxo9	42.43	Lgr5	21.53
1700008O03Rik	11.67	Cpa6	5.08	Lrp5	41.65	Pitx2	21.34
Mterf1b	11.53	Car2	5.04	Tspan8	38.35	Rbm25	20.85
Mterf1a	11.47	Bbx	4.99	Tspoap1	37.30	Nfkbiz	20.46
Lrp5	11.20	Nudcd2	4.95	A630095N17	37.18	Sez6	20.31
Sh3yl1	11.01	Herc1	4.93	Adgb	35.58	Ahnak	20.18
Gm6309	10.36	Coa6	4.90	Rasgrf1	35.32	Trip10	19.87
Arhgef28	10.34	Xpnpep1	4.89	Ssu72	34.03	Pamr1	19.75
4930449I24Rik	10.29	Cisd2	4.83	Stat4	33.66	Pdzrn3	19.50
Gm6408	10.29	Iqgap2	4.64	Bnc2	33.12	Atad1	19.40
Mia2	10.24	Gnas	4.63	Nutm2	33.11	Dscaml1	18.77
Syt10	10.15	Usp25	4.59	Bbs2	32.10	Fam221a	18.74
Tmem184c	9.99	Speer4f2	4.56	Mbd2	30.97	Sft2d1	18.44
Slc35g2	9.73	Ahnak	4.55	Tmeff2	30.73	Nfe2l2	18.41
Zmym6	9.57	Dck	4.51	Arhgef33	30.68	Zfp1	18.37
Ccdc178	9.27	Bbs5	4.51	Galnt18	30.16	Smchd1	18.34
Sparcl1	9.19	Ddx10	4.46	Fra10ac1	30.10	Ppp1r12c	18.30
Mtbp	8.19	Camkmt	4.45	Zfp608	29.68	Zfp292	18.24
Tmem165	7.93	Zfp85	4.43	Phf12	29.03	Kit	18.00
Myocos	7.45	Sec23a	4.39	Nup98	28.97	Lemd3	17.79
M1ap	7.41	Dmc1	4.35	Nr1d1	28.92	Stk32c	17.51
Upp2	7.02	Gm3327	4.33	1110059E24I	28.48	Sfrp2	17.43
Gm5114	6.95	Cntrob	4.31	Med6	28.42	Bcl6	17.20
Ppp1r21	6.76	Tesk2	4.31	Cspp1	28.38	Etv1	17.18
Fastkd3	6.52	Eif4e3	4.27	Tmem165	27.99	Rbl1	17.12
Auh	6.30	Minpp1	4.22	Lipe	27.69	Usp42	16.80
Rab18	6.18	Chek1	4.21	Mki67	27.26	Cul1	16.34
Gm9758	6.11	Klhl7	4.21	Tec	26.95	Ncapd3	16.32
Gm17019	6.02	Siah1a	4.21	Cdk12	26.92	Ptprk	16.30
Clta	6.02	Dhx57	4.20	Sin3a	26.91	Fnip1	16.25
Filip1	6.00	Pax6	4.16	Nxf1	26.82	Trappc10	16.24

Fig. S2. mRNA targets of *A1cf* and *Pum1* RNA-binding proteins. The RNA binding motifs, WUAAUUR for *A1cf* and UGUANAUA for *Pum1*, were used to survey genes expressed in haploid spermatids. The lists include genes with the top 100 ranked position weight matrix (PWM) scores for *A1cf* and *Pum1*. PWM is commonly used to assigns scores to potential target sequences and reflects binding energy of a factor to a particular stretch of nucleotides. The higher the score the more likely the sequence represents a binding target.

Gene family	Gene	Genoinformativity	Mendelian segregation	Gene family	Gene	Genoinformativity	Mendelian segregation
Apobec	*Apobec1*	not detected	**deviation**	***Eif2***	*Eif2a*	non-GIM	ns
	Apobec2	**GIM**	no data		*Eifak1*	non-GIM	ns
	Apobec3	not detected	no data		*Eifak2*	non-GIM	ns
					Eifak4	non-GIM	no data
Eif4	*Eif4a1*	non-GIM	no data		***Eif2b1***	**GIM**	no data
	Eif4a2	not detected	no data		*Eif2b2*	non-GIM	ns
	Eif4b	non-GIM	ns		***Eif2b4***	non-GIM	**deviation**
	Eif4e	**GIM**	**deviation**		*Eif2b5*	non-GIM	ns
	Eif4e2	non-GIM	no data		*Eif2d*	remaining gene	ns
	Eif4e3	**GIM**	no data		*Eif2s1*	non-GIM	ns
	Eif4ebp1	**GIM**	no data		***Eif2s2***	**GIM**	ns
	Eif4ebp2	non-GIM	no data				
	Eif4enif1	non-GIM	ns	***Eif3***	*Eif3a*	not detected	no data
	Eif4g1	**GIM**	no data		*Eif3b*	non-GIM	no data
	Eif4g2	non-GIM	ns		*Eif3d*	not detected	no data
	Eif4g3	**GIM**	no data		*Eif3e*	non-GIM	no data
	Eif4h	remaining gene	**deviation**		*Eif3f*	non-GIM	ns
					Eif3g	not detected	no data
Ppp	*Ppp1ca*	remaining gene	no data		*Eif3h*	non-GIM	ns
	Ppp1r8	not detected	no data		*Eif3l*	non-GIM	no data
	Ppp2cb	not detected	**deviation**		***Eif3j1***	**GIM**	no data
					Eif3k	non-GIM	no data
Shmt	*Shmt1*	non-GIM	ns		*Eif3m*	non-GIM	no data
	Shmt2	not detected	ns				

Fig. S3. Gene families with evidence for GIMs or deviations from Mendelian segregation. These data are presented as a heuristic. Genoinformativity classifications are from Bhutani et al. [5]. and segregation data are from IMPC (mousephenotype.org) or the primary literature. Table S5 provides details about deviations from Mendelian segregation. We used the following criteria to include a gene family here: (1) at least one gene is a GIM, (2) at least one shows fertilization bias, or (3) at least one member of the gene family interacts with a GIM or a bias gene (uniport.org, string-db.org, genecards.org). Interactions can be protein-protein or functional dependency found in mice with a mutant gene, e.g., *Apobec1* and *Dnd1*[6]. There are important caveats: data are from different tissues, species, and conditions; a systematic study under uniform conditions is needed. Genes that are GIMs or show deviations from Mendelian segregation are highlighted in red. 'not detected' – transcripts numbers below the level of detection[5]., 'ns – not significant; 'no data' – segregation has not been tested or reported.

References

1 Lambert, R. Breeding strategies for maintaining colonies of laboratory mice. *TJ Laboratory* (2009).

2 Blanc, V. *et al.* Targeted deletion of the murine apobec-1 complementation factor (acf) gene results in embryonic lethality. *Mol Cell Biol* **25**, 7260-7269, doi:10.1128/MCB.25.16.7260-7269.2005 (2005).

3 Carouge, D. *et al.* Parent-of-origin effects of A1CF and AGO2 on testicular germ-cell tumors, testicular abnormalities, and fertilization bias. *Proc Natl Acad Sci U S A* **113**, E5425-5433, doi:10.1073/pnas.1604773113 (2016).

4 Nikolaou, K. C. *et al.* The RNA-Binding Protein A1CF Regulates Hepatic Fructose and Glycerol Metabolism via Alternative RNA Splicing. *Cell Rep* **29**, 283-300 e288, doi:10.1016/j.celrep.2019.08.100 (2019).

5 Bhutani, K. *et al.* Widespread haploid-biased gene expression enables sperm-level natural selection. *Science* **371**, doi:10.1126/science.abb1723 (2021).

6 Nelson, V. R., Heaney, J. D., Tesar, P. J., Davidson, N. O. & Nadeau, J. H. Transgenerational epigenetic effects of the Apobec1 cytidine deaminase deficiency on testicular germ cell tumor susceptibility and embryonic viability. *Proc Natl Acad Sci U S A* **109**, E2766-2773, doi:10.1073/pnas.1207169109 (2012).
